# Supplementary material for: Effect of an Intensive Lifestyle Intervention on Circulating Biomarkers of Atrial Fibrillation-Related Pathways among Adults with Metabolic Syndrome: Results from a Randomized Trial
Source: J Clin Med. 2024 Apr 7;13(7):2132. doi: 10.3390/jcm13072132 (PMC11012583; doi:10.3390/jcm13072132)
Supplement: Supplementary file 1 [file jcm-13-02132-s001.zip › jcm-2897218-supplementary.pdf]

Supplemental Table S1. *Medication* mainly *use*d by people with metabolic syndrome *in intervention groups at baseline, years 3 and 5*

	Baseline			Year 3			Year 5		
	ILI	control	p-value	ILI	control	p-value	ILI	control	p-value
Antihypertensi*ve drugs*	76.7	76.7	1.00	80.8	77.6	0.37	84.2	84.6	0.92
Lipid-lowering *drugs*	49.6	51.9	0.60	51.3	51.3	0.998	49	51.9	0.51
Oral antidiabetic drugs	21.8	21.8	1.00	26.3	29.7	0.38	35.7	36.1	0.93

**Chi-square test was used to test for significant differences between ILI and control groups.*

Supplemental Table S2. Effect of an intensive lifestyle intervention (ILI) on selected circulating biomarkers stratified by sex and age, PREDIMED-Plus trial

		Difference ILI - control		
		Y3 vs baseline	Y5 vs baseline	p-value for interaction
Male (n=318)	PICP	-4% (-12%, 6%)	-5% (-14%, 5%)	
	hs-TnT	-4% (-9%, 3%)	-2% (-8%, 4%)	
	hsCRP	-15% (-31%, 6%)	-25% (-40%, -7%)	
	3-NT	-12% (-25%, 3%)	-12% (-25%, 3%)	
	NT-proBNP	-5% (-19%, 11%)	-20% (-32%, -5%)	
Female (n=214)	PICP	2% (-11%, 15%)	3% (-9%, 18%)	0.95
	hs-TnT	1% (-6%, 8%)	-4% (-11%, 3%)	0.62
	hsCRP	-1% (-19%, 21%)	7% (-13%, 32%)	0.46
	3-NT	-7% (-22%, 11%)	-22% (-35%, -7%)	0.27
	NT-proBNP	4% (-17%, 30%)	0% (-21%, 26%)	0.58
Age <65 (n=236)	PICP	-1% (-11%, 9%)	-5% (-14%, 5%)	
	hs-TnT	-4% (-11%, 3%)	-1% (-8%, 6%)	
	hsCRP	-13% (-31%, 10%)	-22% (-38%, -2%)	
	3-NT	-9% (-24%, 11%)	-10% (-25%, 9%)	
	NT-proBNP	-11% (-28%, 9%)	-12% (-29%, 8%)	
Age >=65 (296)	PICP	-1% (-12%, 11%)	1% (-10%, 14%)	0.78
	hs-TnT	1% (-5%, 7%)	-4% (-9%, 2%)	0.18
	hsCRP	-7% (-24%, 14%)	-7% (-25%, 15%)	0.99

3-NT	-11% (-23%, 3%)	-21% (-32%, -8%)	0.37
NT-proBNP	9% (-9%, 29%)	-11% (-25%, 6%)	0.49

Notes: Mixed models were used. Results are shown as estimate (95% confidence interval). All the concentrations of biomarkers were log-transformed. Percent changes in concentrations of biomarkers comparing intervention and control arm were presented, calculated as [exp(coefficient)-1]*100. Follow-up exams occurred in years 3 and 5. PICP: C-terminal propeptide of procollagen type I; hsTnT: high sensitivity troponin T; hsCRP: high-sensitivity C reactive protein; 3-NT: 3-nitrotyrosine; NT-proBNP: N-terminal propeptide of B-type natriuretic peptide.

Supplemental Table S3. Changes in log-transformed concentrations of biomarkers through 3 and 5 years according to the intervention group and follow-up time, missing value imputed by multiple imputation by chained equation (MICE), PREDIMED-Plus trial

	Difference ILI - control	
	Y3 vs. baseline	Y5 vs. baseline
PICP	0% (-8%, 9%)	-1% (-9%, 8%)
hsTnT	-1% (-6%, 5%)	-3% (-9%, 2%)
hsCRP	-6% (-20%, 10%)	-12% (-25%, 5%)
3-NT	-8% (-20%, 5%)	-14% (-25%, -1%)
NT-proBNP	1% (-13%, 16%)	-11% (-24%, 3%)

Notes: Mixed models were used. Results are shown as estimate (95% confidence interval). All the concentrations of biomarkers were log-transformed. Percent changes in concentrations of biomarkers comparing intervention and control arm were presented, calculated as [exp(coefficient)-1]*100. PICP: C-terminal propeptide of procollagen type I; hsTnT: high sensitivity troponin T; hsCRP: high-sensitivity C reactive protein; 3-NT: 3-nitrotyrosine; NT-proBNP: N-terminal propeptide of B-type natriuretic peptide.

Supplemental Table S4. Changes in log-transformed concentrations of biomarkers through 3 and 5 years according to the intervention group and follow-up time, adjust for covariates at baseline, PREDIMED-Plus trial

	Difference ILI - control	
	Y3 vs. baseline	Y5 vs. baseline
PICP	-1% (-9%, 7%)	-1% (-8%, 7%)
hsTnT	-2% (-6%, 3%)	-2% (-7%, 3%)
hsCRP	-9% (-22%, 6%)	-13% (-26%, 2%)
3-NT	-7% (-18%, 5%)	-13% (-23%, -2%)
NT-proBNP	-3% (-15%, 11%)	-13% (-24%, 0%)

Notes: Mixed models were used, adjusting for sex, age, education level, marital status, smoking, Mediterranean diet adherence score, and total physical activity at baseline. Results are shown as estimate (95% confidence interval). All the concentrations of biomarkers were log-transformed. Percent changes in concentrations of biomarkers comparing intervention and control arm were presented, calculated as [exp(coefficient)-1]*100. Follow-up exams occurred in years 3 and 5. PICP: C-terminal propeptide of procollagen type I; hsTnT: high sensitivity troponin T; hsCRP: high-sensitivity C reactive protein; 3-NT: 3-nitrotyrosine; NT-proBNP: N-terminal propeptide of B-type natriuretic peptide.

Supplemental Table S5. Changes in log-transformed concentrations of biomarkers through 3 and 5 years according to the intervention group and follow-up time, adjust for eGFR, PREDIMED-Plus trial

	Difference ILI - control	
	Y3 vs baseline	Y5 vs baseline
PICP, mg/mL	-1% (-9%, 7%)	-2% (-9%, 7%)
hs-TnT, ng/L	-2% (-6%, 3%)	-2% (-7%, 2%)
hsCRP, mg/dL	-10% (-23%, 4%)	-14% (-26%, 1%)
3-NT, nM	-11% (-21%, 1%)	-16% (-26%, -5%)
NT-proBNP, pg/mL	-2% (-14%, 13%)	-11% (-22%, 2%)

Notes: Mixed models were used. Results are shown as estimate (95% confidence interval). All the concentrations of biomarkers were log-transformed. Percent changes in concentrations of biomarkers comparing intervention and control arm were presented, calculated as [exp(coefficient)-1]*100. Follow-up exams occurred in years 3 and 5. PICP: C-terminal propeptide of procollagen type I; hsTnT: high sensitivity troponin T; hsCRP: high-sensitivity C reactive protein; 3-NT: 3-nitrotyrosine; NT-proBNP: N-terminal propeptide of B-type natriuretic peptide.
